# 4H-SiC Schottky Barrier Diodes for Efficient Thermal Neutron Detection

**DOI:** 10.3390/ma14175105

**Published:** 2021-09-06

**Authors:** Robert Bernat, Luka Bakrač, Vladimir Radulović, Luka Snoj, Takahiro Makino, Takeshi Ohshima, Željko Pastuović, Ivana Capan

**Affiliations:** 1 Ruđer Bošković Institute, Bijenička Cesta 54, 10000 Zagreb, Croatia; rbernat@irb.hr (R.B.); luka.bakrac@irb.hr (L.B.); 2 Jožef Stefan Institute, Jamova Cesta 39, 1000 Ljubljana, Slovenia; vladimir.radulovic@ijs.si (V.R.); luka.snoj@ijs.si (L.S.); 3 National Institutes for Quantum and Radiological Science and Technology, 1233 Watanuki, Takasaki 370-1292, Japan; makino.takahiro@qst.go.jp (T.M.); ohshima.takeshi@qst.go.jp (T.O.); 4 Australian Nuclear Science and Technology Organisation, 1 New Illawarra Rd., Lucas Heights, NSW 2234, Australia; zkp@ansto.gov.au

**Keywords:** silicon carbide, radiation detector, neutron radiation, nuclear reactor

## Abstract

In this work, we present the improved efficiency of 4H-SiC Schottky barrier diodes-based detectors equipped with the thermal neutron converters. This is achieved by optimizing the thermal neutron converter thicknesses. Simulations of the optimal thickness of thermal neutron converters have been performed using two Monte Carlo codes (Monte Carlo N–Particle Transport Code and Stopping and Range of Ions in Matter). We have used ^6^LiF and ^10^B_4_C for the thermal neutron converter material. We have achieved the thermal neutron efficiency of 4.67% and 2.24% with ^6^LiF and ^10^B_4_C thermal neutron converters, respectively.

## 1. Introduction

Neutron detection is an integral part of the global effort to prevent the propagation as well as the illicit trafficking of nuclear material (U-233, U-235, and Pu-239) at international border crossings. Due to the global shortage of He-3, a detection medium in a gas-filled proportional counter, semiconductor-based detectors have been recognized as a promising alternative. Recently, SiC has attracted a lot of attention as an active material in semiconductor-based neutron radiation detectors [1,2,3,4]. 

We have recently demonstrated that simple detection devices based on 4H-SiC Schottky barrier diodes (SBDs), equipped with the thermal neutron converter films, have a measurable neutron response, which varies linearly with the incident neutron flux [1,5]. Moreover, the SiC-based detector response to various alpha emitters demonstrates an excellent linear behavior as a function of detector’s active area [6]. 

In this work, we present enhancement of the detector system design, which led to maximizing the neutron detection sensitivity of 4H-SiC SBD detector. We optimized the thickness of a thin neutron converter film to obtain the sufficiently high reaction probability combined with the high reaction product escape probability. The optimized converter layer is paired with the 4H-SiC SBD in a robust detector assembly, which can be vacuumed to maximize the detection efficiency of charged reaction products. 

As already described in our previous work [6], a typical 4H-SiC-based detector has the structure of a SBD. Since neutrons can not be directly detected, their presence is deduced from detection of ionizing neutron reaction products, such as alpha particles and tritons. Detailed specifications of SiC-based detectors have been reported elsewhere by several authors [7,8,9].

The efficient thermal neutron detection can be achieved by using a converter layer rich in isotopes with large cross-section for neutrons with energy in the range of k_B_T at room temperature (with k_B_ representing the Boltzmann constant). Possible choices are ^6^Li, ^10^B, ^155^Gd, ^157^Gd, ^235^U, and ^239^Pu. For a thermal neutron with kinetic energy E_n_ = 0.0253 eV (which corresponds to a velocity of 2200 m/s), its respective absorption cross-section is σ_a_ = 940, 3800, 61,000, 255,000, 681, and 1017 barn [10,11,12]. Gadolinium is not used for two reasons: it is extremely rare and reaction products are low-energetic gamma rays—^155^Gd (n, γ) and ^157^Gd (n, γ). Reaction products of interest for neutron detection are mainly low-energy-conversion electrons, mostly grouped around 70 keV. 

Overall, detectors with either ^10^B or ^6^LiF are preferred mainly because the energetic charged-particle reaction products are much easier to discriminate from background radiations. 

The findings of this work are based on the two well-known reactions:

L6i+n→α (2.05 MeV)+H3 (2.73 MeV);Q=4.78 MeVB10+n→α (1.47 MeV)+L7i*(0.84 MeV);Q=2.31 MeVB10+n→α (1.78 MeV)+L7i(1.01 MeV);Q=2.79 MeV 

where ^7^Li^∗^ represents an excited state of ^7^Li (94%), and ^7^Li represents the ground state (6%).

The thickness of the neutron converter plays a crucial role in the detector efficiency. The maximum achievable efficiency, for detectors with single converter film positioned in front of the detector, peaks at 4.4% for the ^6^LiF converter film and 4% for the ^10^B converter film [13,14,15]. Elemental ^6^Li offers the highest achievable efficiency of 11.5%, but it is readily avoided because of its reactivity and flammability, among other properties.

## 2. Materials and Methods

### 2.1. H-SiC Schottky Barrier Diodes

n-type SBDs were produced on nitrogen-doped (up to 5 × 10^14^ cm^−3^) 4H-SiC epitaxial layers, approximately 25 µm thick [1]. The epitaxial layer was grown on the silicon face (8° off) of 350 µm thick silicon carbide substrate without the buffer layer. The Schottky barrier was formed by thermal evaporation of nickel through a metal mask with patterned square apertures of 3 mm × 3 mm, while Ohmic contacts were formed on the backside of the silicon carbide substrate by nickel sintering at 950 °C in the Ar atmosphere.

### 2.2. Electrical Characterization of 4H-SiC SBD’s

The 4H-SiC SBDs were characterized by temperature-dependent current–voltage (I–V) and capacitance–voltage (C–V) measurements, before and after the radiation tests. Measurements were carried out using Keithley 4200 Semiconductor characterization system (Keithley Instruments, Cleveland, OH, USA.

The depth profile of free carriers’ concentration, N*, was determined from capacitance curves using $1/C^{2}$ vs. V relationship, and by calculating $d\left(1/C^{2}\right)/\mathrm{dV}$ as a function of voltage.

### 2.3. Monte Carlo Simulations

Simulations of the effect of the thermal neutron converter thickness on the detector response were performed using Monte Carlo-based MCNP6.2 (Monte Carlo N-Particle Transport Code) and SRIM (Stopping and Range of Ions in Matter) [16,17] codes.

Using MCNP6.2 code, the energy deposition of alpha and triton particles in the active SiC volume was calculated using the F6 tally (averaged energy deposition over a cell). Signal-to-noise ratio and system resolution are strongly related to the energy deposition of reaction products in the SiC. Simulations were run with different neutron converters.

Results of SRIM and MCNP6.2 simulations show that the highest energy deposition rates are achieved with ^10^B_4_C thermal neutron converter films with a thickness of 2.6–3.14 µm and with ^6^LiF film that is 33 µm thick. Table 1 shows that there is good correlation of simulation data with both Monte Carlo codes.

### 2.4. Thermal Neutron Converters

Evaporation material, ^6^LiF, was obtained as a powder, enriched in ^6^Li at 95%, and then evaporated under vacuum onto two different substrates (soda-lime glass—10 mm diameter and 1.4 mm thickness, and aluminum (AlMgSi0.5)—10 mm diameter and 0.5 mm thickness). ^6^LiF was evaporated from Al_2_0_3_ crucible by tantalum heater. The pressure was kept in the range 5 × 10^−5^–2 × 10^−4^ mbar. The evaporation progress was monitored by means of the change in the oscillation frequency of a quartz crystal as the ^6^LiF was evaporated. Leybold Univex 300 thermal evaporation system (Leybold, Cologne, Germany) was used for thin film deposition. 

Thin films of ^10^B_4_C were prepared by magnetron sputtering using a Kurt J. Lesker CMS-18 Sputtering system (Kurt J. Lesker, Jefferson Hills, PA, USA) and a ^10^B_4_C target, 76.5 mm in diameter, 3.2 mm thick, indium-bonded on a copper plate. The target was manufactured by RHP Technology (Seibersdorf, Austria). The mass fraction of isotope ^10^B is 96%. The ^10^B_4_C films were deposited on Al and Si substrate, dimensions 20 mm × 20 mm × 0.35 mm for the Al substrate and approximately 20 mm × 30 mm × 1.0 mm for the Si substrate.

### 2.5. Detector Read-Out System

For the 4H-SiC-based radiation detector read-out system, we used the following electrical components: charge-sensitive preamplifier (CREMAT CR-110, CREMAT, West Newton, MA, USA), Gaussian shaping amplifier (CREMAT CR-200-1µs, CREMAT, West Newton, MA, USA), and multichannel analyzer (AMPTEK MCA 8000D, AMPTEK, Bedford, MA, USA). Electrical power to the system was provided by a battery-powered power supply in order to minimize the level of electronic noise. The reverse bias was applied to detectors using a high-voltage DC-to-DC converter (XP Power CA05P-5, Mouser Electronics, Mansfield, TX, USA), also powered by the standalone power supply. The shaping time was 1.0 μs, and the reverse bias voltage was 100 V.

## 3. Results

**Figure 1:**
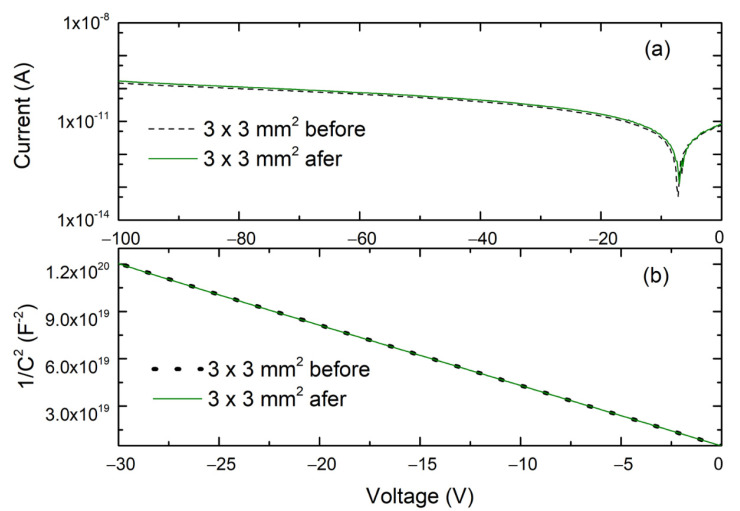
(**a**) I–V characteristics and (**b**) 1/C^2^–V characteristics for the 4H-SiC SBD, before and after radiation test.

The 4H-SiC SBDs were characterized by I–V and C–V measurements (Figure 1), before and after the irradiation tests. All selected SBDs showed excellent rectifying characteristics. The measured ideality factor, *n*, was 1.02 and 1.03 before and after the irradiation tests, respectively. The free carrier concentration was uniform across the whole tested depth, and it was around 5 × 10^14^ cm^−3^.

As shown in Figure 1, thermal neutron radiation tests did not introduce any change in the free carrier concentration.

### Neutron Response of the Detectors

Testing the response of the 4H-SiC prototype detector to thermal neutrons was carried out in the JSI TRIGA reactor. More details are given elsewhere [13]. The Dry Chamber is an irradiation chamber within the concrete reactor body, suitable for the radiation hardness testing of detectors and electronic components [18,19]. The detectors and preamplifiers were installed in the Dry Chamber, and signal and power cables were routed to the reactor platform, where the remaining components were located.

Neutron flux, φ_tot,_ in the Dry Chamber of JSI TRIGA reactor at 250 kW is 1.6 × 10^7^ n cm^−2^ s^−1^. Neutron flux within the neutron energy interval of 0–5 eV, φ_0–5eV,_ is 8.8 × 10^6^ n cm^−2^ s^−1^, relevant for the ^6^Li(n, α)^3^H and ^10^B(n, α)^7^Li reactions.

For measurements, we used a 4H-SiC detector with the active area of 3 mm × 3 mm in combination with the following thermal neutron converters: ^6^LiF (1.4 µm, 2.94 µm, 3.96 µm, 7.24 µm, 9.12 µm, and 26.54 µm) and ^10^B_4_C (1.0 µm, 1.4 µm, 2.0 µm, and 3.0 µm). Each pair of detectors and converters was placed in a custom-designed vacuum-sealed casing maintaining a low vacuum of *p* = 1 × 10^−3^ bar. 

**Figure 2:**
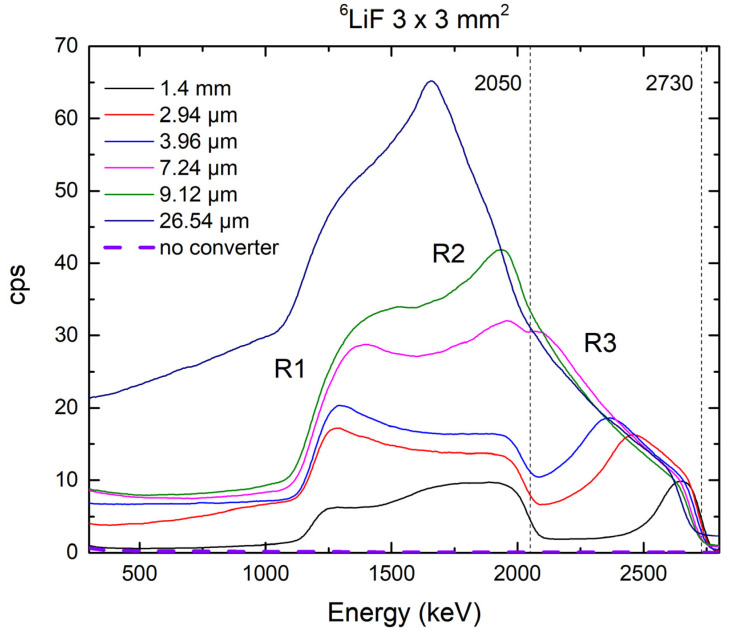
Response of the 4H-SiC SBD detector (3 mm × 3 mm) equipped with ^6^LiF thermal neutron converter layers with different thicknesses to the neutron field of the JSI TRIGA reactor at 250 kW.

Figure 2 shows the response of the 4H-SiC SBD detector equipped with ^6^LiF thermal neutron converters with different thicknesses to the neutron field at a reactor power of 250 kW.

Two maxima are expected, one for alpha particles at 2050 keV and the other for tritons at 2730 keV. Those maxima are clearly visible from our spectra. There is an excellent correlation with simulated data. From the simulation data, we obtained the range for alpha particles of 5.82 μm and for tritons of 32.5 μm in ^6^LiF. For the ^6^LiF thermal neutron converters of 7.24 μm and thicker, we can observe that there is no longer an alpha peak as the particles are fully absorbed by the film. With the film thickness increasing, the shape of spectra is changing as well, mainly because of the absorption of alpha particles of lower energy and subsequently lower range than that of tritons.

Although we might expect that the energies of the charged particles from ^6^Li(n, α)^3^H reaction correspond to the energies of the maximum, this is not the case here for all thin film thicknesses. The most ionization happens at the end of the motion of detected charged particle. If we compare the results for the thinnest converter (1.4 µm), with the Monte Carlo simulations (Table 1), it is noticeable that most of the newly formed charged particles stop in the epitaxial layer of the Schottky diode. In Figure 2., we can distinguish three different regions: 1100–1500, 1500–2000, and 2400–2750 keV. The region for the highest energy can be attributed to tritium particles. The curve is Gaussian, the maximum is clearly defined, and the moment when all the energy is deposited in the epitaxial layer corresponds to the tritium energy for the stated reaction. The second region corresponds to the energy of alpha particles, and with the approximation of the Bragg curve, we can also conclude that the moment when all the energy is deposited in the epitaxial layer corresponds to the energy of alpha particles formed by ^6^Li(n, α)^3^H reaction. The first region belongs to the tritium particles or to low-energetic alpha particles. Tritium particles are more likely because throughout the energy range, it is this energy that is gradually deposited, and the shape of the curve corresponds to the Bragg curve for triton particles. 

In the case of the thicker converters, we notice that the first region increases and the second decreases: the layer thickness increases and fewer and fewer alpha particles of maximum energy pass through the converter layer. At a converter thickness of 7.24 µm, we no longer see the end of the Bragg curve for alpha particles, but the second region merges with the third, that is, with that attributed to tritium. This corresponds to our assumptions because at this thickness, all particles of maximum energy should be deposited in the converter layer. By analyzing the response of a 4H-SiC SBD with a converter with maximum thickness, we notice only one region, and it, in combination with Monte Carlo simulations, can be attributed with great certainty to the tritium formed in ^6^Li(n, α)^3^H. The first region continuously grows through all the thicknesses of the converter, and a very high plateau can be noticed when the thickest converter responds. The amplitude of the maximum attributed to tritium particles is higher than the amplitude of the maximum attributed to alpha particles because heavier alpha particles have energy deposited in fewer interactions compared to lighter tritium. Since in the thickest converter there is a full deposition of alpha particle energy in the neutron converter layer, there is no pronounced maximum but a slow loss of energy through the thickness of the thin film is manifested by a high plateau. This interpretation of the spectrum has already been reported earlier in the literature [4,20]. 

**Figure 3:**
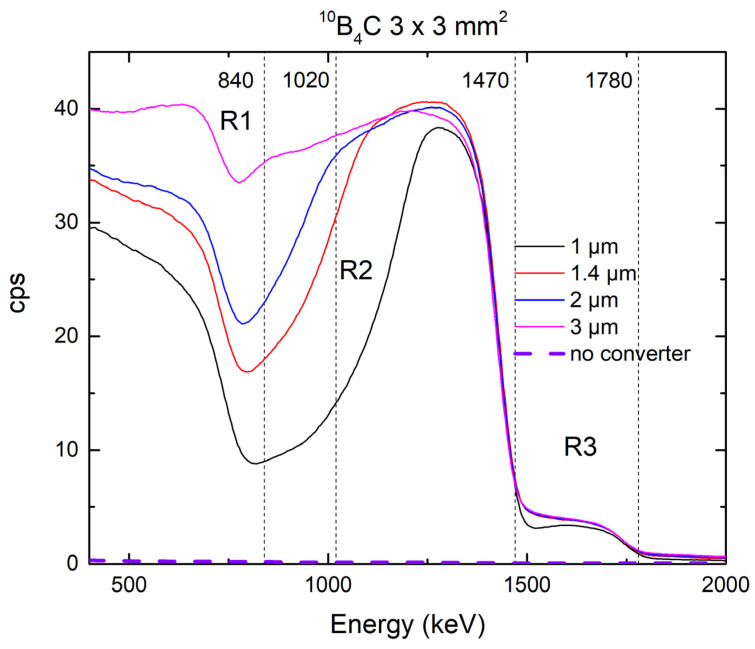
Response of the 4H-SiC SBD detector (3 mm × 3 mm) equipped with ^10^B_4_C thermal neutron converter layers with thicknesses of 1.0, 1.4, 2.0, and 3.0 μm to the neutron field of the JSI TRIGA reactor at 250 kW.

Figure 3 shows the response of the 4H-SiC SBD detector with ^10^B_4_C thermal neutron converters layers with different thicknesses to the neutron field at reactor power of 250 kW.

For the detector with ^10^B_4_C neutron converter, calibration obtained from Am-241 measurement was applied as well. Due to a high ratio for the ^10^B(n, α)^7^Li reaction in the excited state (94%), two maxima are observed as well, one for alpha particles at 1470 keV and the other for ^7^Li at 840 keV. From the simulation data, we obtained the range of alpha particles at 3.14 µm and 1.58 µm for ^7^Li, respectively. For both film thicknesses we can observe a spectral shoulder due to ^7^Li particles and a maximum due to alpha particles. In the spectra of thicker film, there is obvious absorption of the ^7^Li particles as the thickness of 1.58 μm is reached. There is no difference in the height of the alpha peak in two recorded spectra.

The selection of the converter thicknesses was made so that most of the charged particles should pass through the converter and stop in the epitaxial layer of the 4H-SiC SBD. Again, we notice three different regions in which energy maxima occur: 400–850, 850–1500, and 1500–1800 keV). By analyzing the 4H-SiC SBDs’ response, we find both the reaction products for the excited state reaction and one reaction product for the ground state reaction. Due to the low representation of the reaction in the ground state (6%) and the shading of other particles found in this part of the energy spectrum, it is not possible to distinguish the ^7^Li nucleus with an energy of 1.02 MeV. It is interesting to note that the ratio of the amplitude of the maximum, which is attributed to the alpha particles in the ground and excited states, corresponds to the ratio of the representation of individual reactions (6% and 94%). As the thickness of the converter layer increases, the energy maximum of the alpha particles overshadows that of the ^7^Li core, and it is to be expected that a further increase in the layer thickness would show only one maximum, namely, that for alpha particles of 1.47 MeV energy would attenuate all particles formed by transmutation of the converter material. Here, we do not notice an increase in the energy maximum amplitude for alpha particles as a function of the thickness of the converter layer, as was the case with the maximum tritium energy amplitude in the ^6^LiF converter.

We prepared thin film converters of ^6^LiF on both glass (1.4 mm thick) and aluminum (0.5 mm thick) substrates, and we found no influence on the detector response whatsoever.

**Figure 4:**
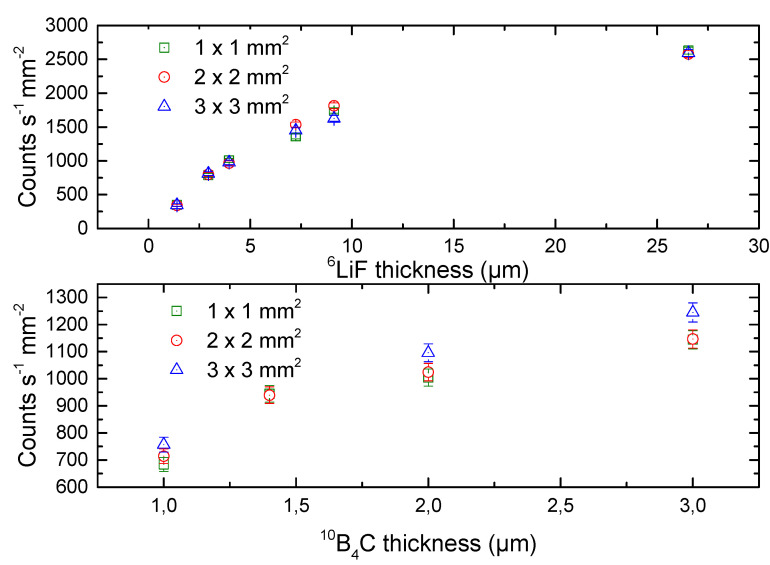
A correlation of thickness for both ^6^LiF and ^10^B_4_C converter layers with normalized surface area of three different detector sizes (1 mm × 1 mm, 2 mm × 2 mm, and 3 mm × 3 mm). The total count rate is shown on the *y*-axis.

To obtain additional information on 4H-SiC detector response, we compared the 4H-SiC SBDs with different active areas. Figure 4 shows a correlation of thickness for both ^6^LiF and ^10^B_4_C converter layers with normalized surface area of three different detector sizes (1 × 1, 2 × 2, and 3 × 3). Yield rate per unit detector area is the function of the ^6^LiF converter layer thickness and does not depend on detector active area (Figure 4) as expected. This is not the case for ^10^B_4_C converter layer (Figure 4), where we observe differences in calculated yield rates for different detector sizes.

The detection efficiency of the device can be simply evaluated starting from the total counts above 500 keV [15,21]. The neutron detection efficiency limitation is a consequence of reaction product self-absorption. Maximum reported efficiency for the single-coated detectors is 5% for the thermal neutrons [13,14,15,21,22,23].

Table 2 shows calculated detector efficiency for different thermal neutron converter thicknesses.

Although the low vacuum in detector–converter casing does not change detector yield, the peak resolution (sharpness, definition) is better in the vacuum compared to no vacuum. Sharper maximums are observed in vacuum, and the spectra is shifted to the higher energies due to the fact that less energy is deposited in the air. The range of charged particles in air is 4.35 mm for the ^7^Li, resulting from the ^10^B(n, α)^7^Li reaction, and up to 63.2 mm for ^3^H, resulting from the ^6^Li(n, α)^3^H. During the irradiations in the Dry Chamber, the distance between the thermal neutron converter film and the active detector surface was not more than 2 mm. In the used detector system, even without vacuum conditions, all the charged particles that were produced in the converters had reached the detector; dissipation of the kinetic energy of the emitted charged particles is not of a particular importance in this case.

## 4. Conclusions

This paper presents findings on development and efficiency optimization of the thermal neutron converter films as an important part of the 4H-SiC SBD neutron detector. In our previous studies [1,6,24,25,26], we have reported results on 4H-SiC material radiation hardness, and response to ionizing particles (alpha and gamma radiation). We have successfully assembled a set of electronic components for read-out, detectors, and thermal neutron converters to develop a fully functional, portable, battery-powered thermal neutron detector. 

Here, we present a newly developed 4H-SiC-based detector prototype with the active surface area of 3 mm × 3 mm equipped with thermal neutron converter films prepared by thermal evaporation (^6^LiF) and magnetron sputtering (^10^B_4_C). The most effective thermal neutron converter films are the thickest ones, 26.54 μm and 3 μm for ^6^LiF and ^10^B_4_C, respectively. There is an evident difference in the response of the detector using the ^6^LiF over the ^10^B_4_C neutron converter (4.67 and 2.24 respectively). The reason for such a deviation is not yet known and will be the subject of our further research.

We have reached the highest reported efficiency [13,14,15,21,22] for the 4H-SiC-based neutron detector of almost 5% for the thermal neutrons.

## Figures and Tables

**Table 1:** Correlation of Monte Carlo simulation codes.

Monte Carlo Code	Simulated Thermal Neutron Converter Thickness for the Highest Range of Selected Particles			
	α in ^6^Li(n, α)^3^H	^3^H in ^6^Li(n, α)^3^H	α in ^10^B(n, α)^7^Li	^7^Li in ^10^B(n, α)^7^Li
MCNP6.2	5.8 µm	33 µm	2.6 µm	*
SRIM	5.82 µm	32.5 µm	3.14 µm	1.58 µm

* Not simulated due to MCNP6.2 limitations for transport of heavy particles.

**Table 2:** 4H-SiC SBD detector (3 mm × 3 mm) efficiency for different thermal neutron converter thicknesses.

Converter	Thickness (μm)	Sensitivity (Counts s^−1^ per n cm^−2^s^−1^)	Efficiency (%)
^10^B_4_C	1.0	1.23 × 10^−3^	1.36
^10^B_4_C	1.4	1.66 × 10^−3^	1.84
^10^B_4_C	2.0	1.78 × 10^−3^	1.97
^10^B_4_C	3.0	2.02 × 10^−3^	2.24
^6^LiF	1.4	5.64 × 10^−4^	0.63
^6^LiF	2.94	1.31 × 10^−3^	1.46
^6^LiF	3.96	1.59 × 10^−3^	1.76
^6^LiF	7.24	2.35 × 10^−3^	2.61
^6^LiF	9.12	2.64 × 10^−3^	2.93
^6^LiF	26.54	4.20 × 10^−3^	4.67

## Data Availability

Data is contained within the article.
